# Information and practices of antenatal colostrum expression: a cross-sectional study of pregnant women in Norway

**DOI:** 10.1186/s12884-026-09035-y

**Published:** 2026-05-21

**Authors:** Maria Flåten, Tina Marie Stokke, Maren Johnsen, Eline Skirnisdottir Vik

**Affiliations:** 1 Faculty of Health Sciences, UiT The Arctic University of Norway, Hansine Hansens veg 18, Tromsø, 9019 Norway; 2 University Hospital of North Norway, Hansine Hansens veg 67, Tromsø, 9019 Norway; 3 Faculty of Health and Social Sciences, Western Norway University of Applied Sciences, Inndalsveien 28, Bergen, 5063 Norway

**Keywords:** Antenatal education, Milk expression, Hand expression, Breast milk, Breastfeeding, Lactation, Pregnancy, Colostrum

## Abstract

**Background:**

Antenatal colostrum expression (ACE) may increase breastfeeding self-efficacy. Although interest in ACE is growing in Western countries, evidence from Norway is scarce. This study examined whether, and in which form, pregnant women receive information about ACE, and explored characteristics of women who practise ACE compared to those who do not.

**Methods:**

We conducted a cross-sectional survey among women who gave birth after 37 + 0 weeks´ gestation in Norway between 1 October 2023 and 30 September 2024. Data were collected through an anonymous online questionnaire (23 October –20 November 2024) from 1640 women and analysed using descriptive statistics, Chi-square, Fisher’s exact test and Mann-Whitney U-test.

**Results:**

Two-thirds of the women received information about ACE before birth. One quarter received information from healthcare personnel, and twice as many from other sources. The information from healthcare personnel was mainly given by midwives, in oral form. Women who expressed antenatal colostrum were more likely to have heard about ACE before pregnancy (*p* < 0.001), to have received information from healthcare personnel (*p* < 0.001) and other sources (*p* < 0.001), and to have been advised by healthcare personnel to practise ACE (*p* < 0.001), compared to those who had not undertaken ACE. Expressing antenatal colostrum correlated with being younger (*p* < 0.001) and primiparous (*p* < 0.001). The primary reason for practising ACE was to avoid formula, while non-practice was most often attributed to lack of knowledge.

**Conclusions:**

Women who received information about ACE primarily obtained this information from other sources than healthcare personnel. Women should have access to evidence-based information about ACE, including potential benefits and disadvantages, to make informed decisions. This underscores the need for national guidelines and further research on ACE and its impact on breastfeeding outcomes.

**Supplementary Information:**

The online version contains supplementary material available at 10.1186/s12884-026-09035-y.

## Background

The World Health Organization (WHO) recommends exclusive breastfeeding for the first six months of life, with continued breastfeeding up to two years of age or beyond [1]. Breastfeeding and breast milk are well documented to offer a range of health benefits for both mother and child [2, 3]. Antenatal colostrum expression (ACE) may be practised by pregnant women as part of their preparation for breastfeeding [4]. ACE refers to the manual expression of colostrum during pregnancy, typically initiated from around gestational week 36–37, with the aim of collecting and storing colostrum for potential use after birth [5]. The practice may be particularly useful when there is a suspicion that the newborn could require supplementary feeding after birth [6, 7]. In several Western countries, there is growing interest in ACE among both pregnant women and healthcare personnel [5, 8, 9]. Most research on the topic has been published over the past decade, highlighting both potential benefits and limitations [4, 10].

A systematic review which included ten studies from three Western countries found that ACE may enhance empowerment, motivation and confidence related to breastfeeding, and reduce formula use in maternity wards [4]. Two of the included studies suggested that the practice could support breastfeeding by increasing breastfeeding rates 24 h after birth or at discharge [11, 12], while another found no such association [13]. Some women described the practice as uncomfortable and time-consuming, and expressed concerns about triggering contractions or preterm birth [4]. Others experienced a sense of failure if they were unable to express colostrum during pregnancy, which led to concerns about insufficient milk production after birth. Nevertheless, many women found the practice feasible and reassuring, and the successful expression of colostrum contributed to greater confidence in their ability to breastfeed [4].

Another recent systematic review which included 22 studies from six Western countries found that both pregnant women and healthcare personnel expressed uncertainty about ACE [10]. Some women attributed this to their own lack of knowledge, either being unaware that ACE was possible or lacking information about its potential advantages and disadvantages [10]. Others reported that some healthcare personnel were unfamiliar with the practice, discouraged the use of antenatally expressed colostrum, failed to store brought-in colostrum properly or discarded it [10]. These findings underscore the need for greater knowledge dissemination, targeting both pregnant women and their partners, as well as healthcare personnel involved in antenatal, intrapartum and postpartum care.

Previous research has primarily explored the relationship between ACE and breastfeeding prevalence, exclusive breastfeeding and neonatal hypoglycaemia, as well as the feasibility of the practice [9, 11, 12, 14–16]. Safety has also been studied through outcomes such as admissions to neonatal intensive care units and gestational age at birth [9, 11]. The majority of the research has focused on relatively homogeneous populations, particularly women with diabetes during pregnancy [11, 12, 14, 16–18]. Additional studies have investigated women’s experiences with ACE [19–24]. Practising ACE is considered safe and has not been shown to be associated with preterm birth or an increase in admissions to the neonatal intensive care unit [8, 9, 11]. However, ACE does not appear to lead to earlier onset of lactogenesis II [18, 25]. There have been conflicting results regarding whether ACE can be associated with increased breastfeeding rates [8, 9, 11, 12, 26] and reduced use of formula [8, 12]. ACE may increase breastfeeding self-efficacy [10]. According to Bandura, self-efficacy refers to an individual’s belief in their capacity to organize and carry out actions required to achieve specific goals, emphasizing perceived ability rather than actual skill level [27]. Individuals with high self-efficacy are more likely to approach challenges as tasks to be mastered, while those with low self-efficacy tend to doubt their capabilities, avoid difficult situations, and are more likely to give up when facing obstacles [27]. Systematic reviews have shown that women with high breastfeeding self-efficacy are more likely to exclusively breastfeed and to breastfeed for a longer duration [28–30].

To our knowledge, no studies have specifically examined the characteristics of pregnant women who practise ACE compared to those who do not. The Swedish Association of Midwives recommends that women with diabetes and otherwise uncomplicated pregnancies be offered information about ACE [31]. However, the practice is not addressed in the Norwegian national clinical guideline for antenatal care [32]. Furthermore, we are not aware of any studies investigating whether pregnant women in Norway receive information about ACE, or how such information is conveyed. This indicates a need for further research to expand knowledge and awareness of the practice.

The aim of this study was to investigate whether, and in which form, pregnant women receive information about ACE, and to explore characteristics of women who engage in ACE compared to those who do not.

## Methods

This is a cross-sectional study.

### Setting

In Norway, antenatal care is a free healthcare service offering regular consultations with midwives or general practitioners. The Directorate of Health recommends exclusive breastfeeding until six months of age, continued breastfeeding until the child is one year old, and beyond if desired by both mother and child [33]. National antenatal guidelines emphasize practical guidance on breastfeeding towards the end of the pregnancy, however they do not mention ACE as a general recommendation. In light of new research [8, 9, 11, 16] and increased attention on the subject different health regions may have local guidelines on ACE practice [34].

### Study sample and recruitment

Eligible participants were women aged 18 or older who had given birth in Norway between 1 October 2023 and 30 September 2024, from gestational week 37 + 0. Women who delivered preterm or were under 18 years of age were excluded. Participants were recruited through self-selection via Facebook, Instagram and Snapchat, in the period from 23 October to 20 November 2024. Recruitment materials consisted of visually designed posts containing a direct link and QR code to the digital questionnaire, which were shared by private persons as well as social media profiles dedicated to pregnancy, childbirth, and the postpartum period, and with substantial followings, on different occasions during the data collection period.

### Questionnaire and variables

A digital, closed-ended questionnaire was developed for the study and included 26 items, divided into three main sections: background characteristics, ACE practice and information received [see Additional file 1]. The questionnaire included items on whether participants had practised ACE (yes, no, other). Participants who had received information from healthcare professionals were asked to specify from whom (midwife, general practitioner, gynecologist, nurse, other), while those who had received information from other sources indicated where they had learned about ACE (social media, influencer, Ammehjelpen (the Norwegian breastfeeding mother-to-mother support group), family member, friend, book, TV program, other). Questions were designed with predefined response options. Some items used Likert scales to assess perceived sufficiency of information. Follow-up questions were routed based on responses to reduce participant burden and ensure relevance. A codebook was generated automatically. Because all questions were mandatory and response options were fixed, the dataset contained no missing or extreme values.

The questionnaire was pilot tested in October 2024 in two rounds to identify potential errors and ensure clarity and feasibility within the target group. Based on feedback, minor revisions were made to the wording of questions and response options, and a brief definition of ACE was added.

### Statistical analyses

Descriptive statistics were used to summarize the distribution of responses across the full sample and within subgroups. Participants were categorized into two groups: those who had practised ACE and those who had not. Frequencies and percentages were calculated for key variables, including age, health region, education level and parity. Cross-tabulations were constructed to compare the groups and to illustrate the proportions of women who received information about ACE and the sources of that information.

Pearson’s chi-square test was used to examine associations between categorical variables and ACE practice. Variables included education level, health region, and parity. When more than 20% of expected cell frequencies were less than five, Fisher’s exact test was applied as a suitable alternative. This was necessary, for example, when analysing the main provider of antenatal care.

To compare perceived sufficiency of information between women who had practised ACE and those who had not, the Mann-Whitney U test was used. This non-parametric test was appropriate for comparing two independent groups when the dependent variable was ordinal. Participants rated the sufficiency of information received from healthcare personnel and other sources on a four-point Likert scale. Rankings were analysed to determine whether significant differences existed in perceived support between the two groups.

Statistical significance was set at *p* < 0.05 for all tests. *P*-values below this threshold were interpreted as evidence of a statistically significant association or group difference. In addition, 95% confidence intervals (CI) were calculated to indicate the precision of the estimates. No a priori sample size or power calculation was performed, as this was a descriptive cross-sectional study.

All analyses were conducted using IBM SPSS Statistics version 29.

### Ethics

The study was conducted in accordance with the Declaration of Helsinki and assessed by the Regional Committee for Medical and Health Research Ethics (REK) prior to recruitment (reference number: 825190). The study was deemed to fall outside the remit of the Norwegian Act on Medical and Health Research. The study was conducted in accordance with UiT The Arctic University of Norway’s guidelines for data management [35]. Data were collected anonymously using the web-based tool *Nettskjema* [36]. In line with UiT The Arctic University of Norway’s guidelines for data management and the purpose of protecting participants’ privacy, detailed personal data were not collected; only categorical background data were included. Informed consent was obtained through voluntary participation: by accessing and completing the anonymous questionnaire, participants consented to take part in the study. After the data collection, the data were stored in Microsoft Teams, a cloud storage service which requires two-factor authentication to log in. Only members of the project group have access to the data, and the data will be deleted when the project period ends.

## Results

**Fig. 1:**
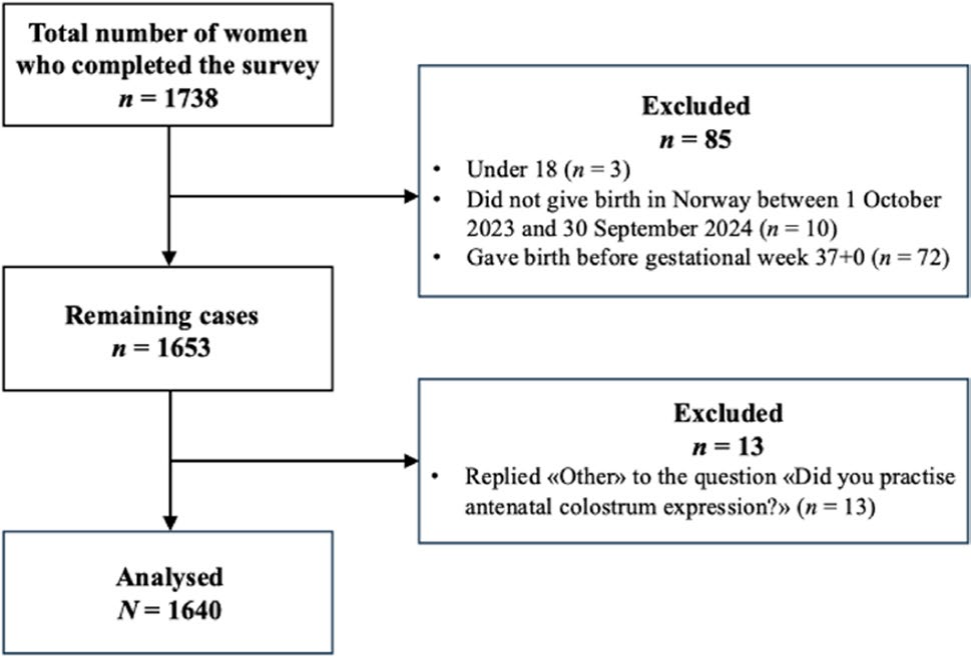
Flowchart of the derivation of the study sample

When data collection ended on 20 November 2024, a total of 1,738 women had completed the survey. Respondents who indicated they were under 18 years of age, had not given birth in Norway between 1 October 2023 and 30 September 2024, or had given birth before gestational week 37 + 0 were excluded, as they did not meet the inclusion criteria. A small group who answered “Other” to the question “Did you practise antenatal colostrum expression?” was also excluded, since it was unclear whether they had practised ACE. The final sample included 1,640 respondents (Fig. 1).

Characteristics of the sample are presented in Table 1 (*N* = 1,640). Nearly half of the participants were in the 30–34 age group (48.5%), and the majority were first-time mothers (55.9%). The health region most frequently reported was South-East Norway (48.0%). A large proportion had completed more than four years of higher education at college or university (46.9%). Fewer women had practised ACE during pregnancy (41.4%) compared to those who had not (58.6%). There was a statistically significant association between practising ACE and being younger (*p* < 0.001) as well as being a first-time mother (*p* < 0.001).

**Table 1:** Characteristics of the participants (*N* = 1640)

Practised ACE^a^	Yes (*n* = 679)	95% CI	No (*n* = 961)	95% CI	*p*-value
	*n* (%)		*n* (%)		
Age					
18–24	39 (5.7)	4.2–7.7%	36 (3.7)	2.7–5.1%	
25–29	223 (32.8)	29.4–36.4%	258 (26.8)	24.1–29.7%	
30–34	333 (49.0)	45.3–52.8%	463 (48.2)	45.0-51.3%	
35–39	75 (11.0)	8.9–13.6%	175 (18.2)	15.9–20.7%	
40 or older	9 (1.3)	0.7–2.4%	29 (3.0)	2.1–4.2%	< 0.001
Education level					
Primary/secondary/sixth form colleges	90 (13.3)	10.9–16.0%	156 (16.2)	14.0–18.7%	
University, 1–4 years	259 (38.1)	34.5–41.8%	366 (38.1)	35.1–41.2%	
University, more than 4 years	330 (48.6)	44.9–52.4%	439 (45.7)	42.5–48.8%	0.22
Health region					
Northern Norway	94 (13.8)	11.4–16.6%	129 (13.4)	11.4–15.7%	
Central Norway	110 (16.2)	13.6–19.1%	189 (19.7)	17.2–22.3%	
Western Norway	145 (21.4)	18.4–24.6%	185 (19.3)	16.9–21.8%	
South-East Norway	330 (48.6)	44.9–52.4%	458 (47.7)	44.5–50.8%	0.31
Parity					
Primipara	426 (62.7)	59.1–66.3%	491 (51.1)	47.9–54.2%	
Multipara	253 (37.3)	33.7–40.9%	470 (48.9)	45.8–52.1%	
Other	0 (0.0)		0 (0.0)		< 0.001

^a^ Antenatal colostrum expression

Two-thirds (67.3%) of the women (*N* = 1,640) received information about ACE during pregnancy, while one-third (32.7%) did not (not shown). Table 2 shows whether women received information about ACE, based on whether they had practised it. Among all participants, roughly one-third (35.4%) were aware of ACE before becoming pregnant. Just over a quarter (28.5%) received information about the practice from healthcare personnel during pregnancy, and one in five (19.4%) had been recommended to do so by healthcare personnel. Compared to women who had not practised ACE, those who had were significantly more likely to report prior awareness of the practice (*p* < 0.001), to have received information from healthcare personnel (*p* < 0.001), whether in municipal services (*p* < 0.001), from private healthcare providers (*p* < 0.001), or at maternity wards or outpatient clinics (*p* < 0.001), to have been recommended the practice by healthcare personnel (*p* < 0.001), and to have received information from sources other than healthcare personnel (*p* < 0.001).

**Table 2:** Information about antenatal colostrum expression (*N* = 1640)

Practised ACE^a^	Yes	95% CI	No	95% CI	*p*-value
	*n* (%)		*n* (%)		
Were you aware of ACE before becoming pregnant?					
Yes	274 (40.4)	36.7–44.1%	306 (31.8)	29.0–34.8%	
No	402 (59.2)	55.5–62.9%	644 (67.0)	64.0–69.9%	
Other	3 (0.4)	0.1–1.2%	11 (1.1)	0.6–2.0%	< 0.001
Who did you mainly visit for prenatal consultations?					
Midwife	494 (72.8)	69.3–76.0%	693 (72.1)	69.2–74.9%	
General practitioner (GP)	22 (3.2)	2.1–4.8%	46 (4.8)	3.6–6.3%	
A combination of services (midwife, GP, maternity wards or outpatient clinics)	162 (23.9)	20.8–27.2%	218 (22.7)	20.1–25.4%	
Did not attend prenatal consultations	0 (0.0)	-	2 (0.2)	0.0–0.7%	
Other	1 (0.1)	0.0–0.7%	2 (0.2)	0.0–0.7%	0.43^b^
Did you receive information about ACE from healthcare personnel?					
Yes	342 (50.4)	46.6–54.1%	126 (13.1)	11.1–15.4%	
No	316 (46.5)	42.8–50.3%	825 (85.8)	83.5–87.9%	
Other	21 (3.1)	2.0–4.6%	10 (1.0)	0.5–1.8%	< 0.001
Did you receive information about ACE during prenatal consultations or courses from healthcare personnel in municipal services?					
Yes	316 (46.5)	42.8–50.3%	132 (13.7)	11.7–16.0%	
No	336 (49.5)	45.7–53.2%	815 (84.8)	82.4–87.0%	
Other	27 (4.0)	2.7–5.6%	14 (1.5)	0.8–2.4%	< 0.001
Did you receive information about ACE during prenatal consultations or courses from private healthcare providers?					
Yes	65 (9.6)	7.5–12.0%	22 (2.3)	1.5–3.4%	
No	325 (47.9)	44.1–51.6%	562 (58.5)	55.3–61.6%	
Did not visit private healthcare providers	279 (41.1)	37.4–44.8%	372 (38.7)	35.7–41.8%	
Other	10 (1.5)	0.8–2.6%	5 (0.5)	0.2–1.1%	< 0.001
Did you receive information about ACE at the maternity ward prior to the birth?					
Yes, during an admission/ultrasound/outpatient appointment at the maternity ward/outpatient clinic prior to the admission					
related to the birth	68 (10.0)	7.9–12.4%	19 (2.0)	1.2–3.0%	
Yes, during the admission related to the birth	19 (2.8)	1.8–4.2%	26 (2.7)	1.8–3.9%	
No, I did not receive information about ACE during an admission/ultrasound/outpatient appointment at the					
maternity ward/outpatient clinic	513 (75.6)	72.2–78.7%	796 (82.8)	80.3–85.1%	
Was not admitted/at an ultrasound or outpatient appointment prior to the birth	68 (10.0)	7.9–12.4%	103 (10.7)	8.9–12.8%	
Other	11 (1.6)	0.9–2.8%	17 (1.8)	1.1–2.8%	< 0.001
Were you recommended by healthcare personnel to practise ACE?					
Yes	283 (41.7)	38.0–45.4%	35 (3.6)	2.6–5.0%	
No	365 (53.8)	50.0–57.5%	907 (94.4)	92.8–95.7%	
Other	31 (4.6)	3.2–6.3%	19 (2.0)	1.2–3.0%	< 0.001
Did you receive information about ACE from other sources than healthcare personnel?					
Yes	487 (71.7)	68.2–75.0%	401 (41.7)	38.6–44.9%	
No	184 (27.1)	23.9–30.5%	545 (56.7)	53.6–59.8%	
Other	8 (1.2)	0.6–2.2%	15 (1.6)	0.9–2.5%	< 0.001

^a^ Antenatal colostrum expression ^b^ Fisher’s exact test

**Fig. 2:**
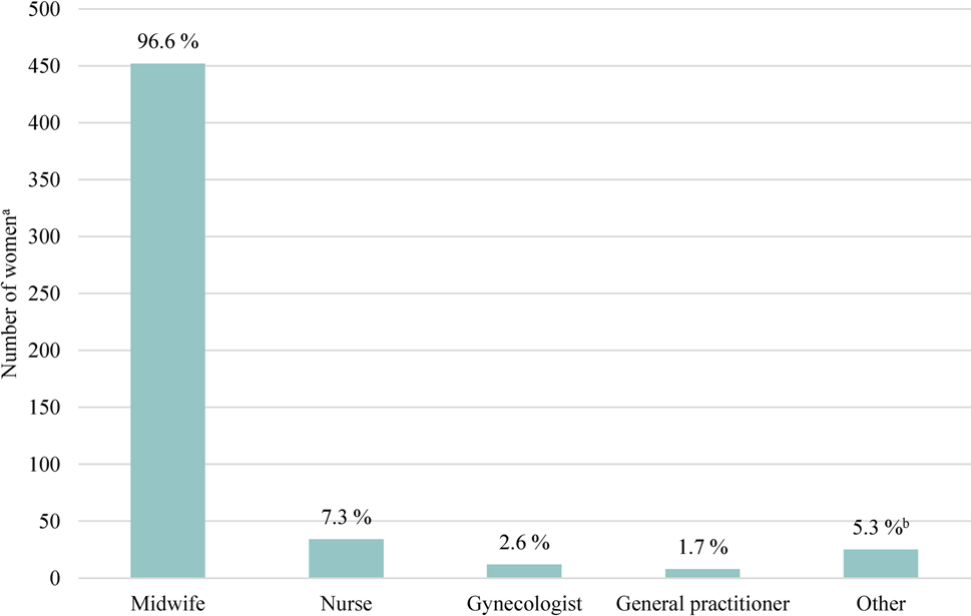
Which type of healthcare personnel women reported receiving information about antenatal colostrum expression from. ^a^ Only participants who responded “Yes” to the question “Did you receive information about antenatal colostrum expression from healthcare personnel?” received this question (*n* = 468). ^b^ Respondents could tick multiple answers. Total percentage therefore exceeds 100%

Figure 2 shows which types of healthcare personnel the women reported receiving information about ACE from. Nearly all respondents who received information from healthcare personnel stated they had received it from a midwife.

**Fig. 3:**
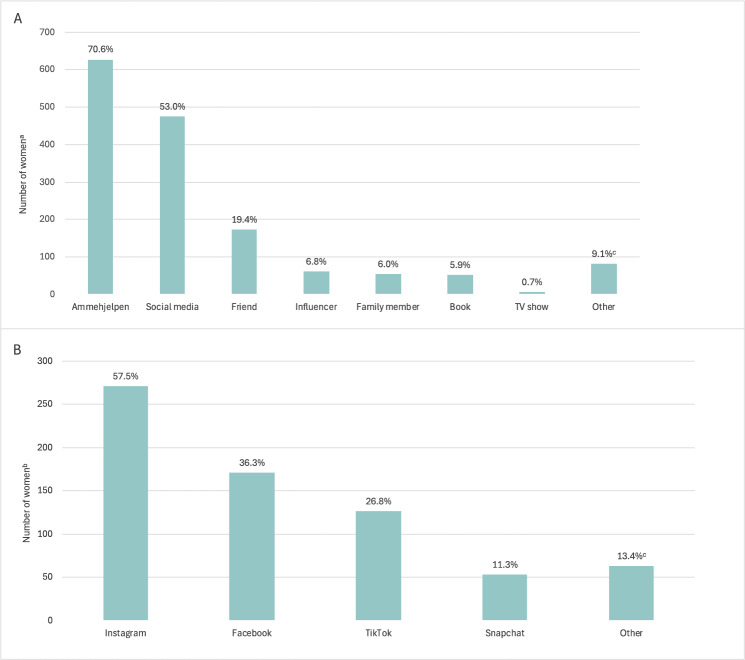
(**A**) Other sources of information about antenatal colostrum expression (**B**) Which social media platforms. a Only participants who responded “Yes” to the question “Did you receive information about antenatal colostrum expression from other sources than healthcare personnel?” received this question (*n* = 888). b Only participants who responded “Social media” to the question “From which sources other than healthcare personnel did you receive information about antenatal colostrum expression?” received this question (*n* = 471). c Respondents could tick multiple answers. Total percentage therefore exceeds 100%

Compared to the number of women who received ACE information from healthcare personnel, twice as many received information from other sources. These women were asked to specify which sources. The responses are illustrated in Figure 3 (A). Most women reported receiving information from Ammehjelpen (the Norwegian breastfeeding mother-to-mother support group), a non-governmental organization, followed by social media. One-quarter had received information from a friend and/or a family member (Fig. 3).

Women who reported having received information from social media were also asked to indicate which platforms. These responses are shown in Figure 3 (B). Most women identified Instagram as their source.

There was a statistically significant difference in how sufficient women felt the information they had received about ACE from healthcare personnel (*p* < 0.001) and from non-healthcare sources (*p* < 0.001) was in helping them potentially carry out the practice. This is shown in Table 3. Women who had practised ACE more often reported that the information received from either source was sufficient or very sufficient, compared to those who had not practised ACE.

**Table 3:** To what extent the women felt that the information they received was sufficient

Practised ACE^a^	Yes	95% CI	No	95% CI	Mann-Whitney U	*p*-value
	*n* (%)		*n* (%)			
To what extent did you feel that the information you received from healthcare personnel was sufficient to be able to potentially practise ACE?^b^						
To a small extent	32 (9.4)	6.6–12.8%	34 (27.0)	19.8–35.2%		
To some extent	100 (29.2)	24.6–34.2%	56 (44.4)	36.0–53.2%		
To a large extent	124 (36.3)	31.3–41.5%	25 (19.8)	13.6–27.4%		
To a very large extent	83 (24.3)	20.0–29.0%	7 (5.6)	2.5–10.6%		
Other	3 (0.9)	0.2–2.3%	4 (3.2)	1.1–7.4%	13430.5	< 0.001
To what extent did you feel that the information you received from other sources than healthcare personnel was sufficient to be able to potentially practise ACE?^c^						
To a small extent	29 (6.0)	4.1–8.3%	128 (31.9)	27.5–36.6%		
To some extent	125 (25.7)	21.9–29.7%	151 (37.7)	33.0–42.5%		
To a large extent	183 (37.6)	33.4–41.9%	80 (20.0)	16.3–24.1%		
To a very large extent	146 (30.0)	26.0–34.2%	29 (7.2)	5.0–10.1%		
Other	4 (0.8)	0.3–1.9%	13 (3.2)	1.8–5.3%	53507.5	< 0.001

^a^ Antenatal colostrum expression. ^b^ Only participants who responded “Yes” to the question “Did you receive information about ACE from healthcare personnel?” received this question (*n* = 468). ^c^ Only participants who responded “Yes” to the question “Did you receive information about ACE from other sources than healthcare personnel?” received this question (*n* = 888)

**Fig. 4:**
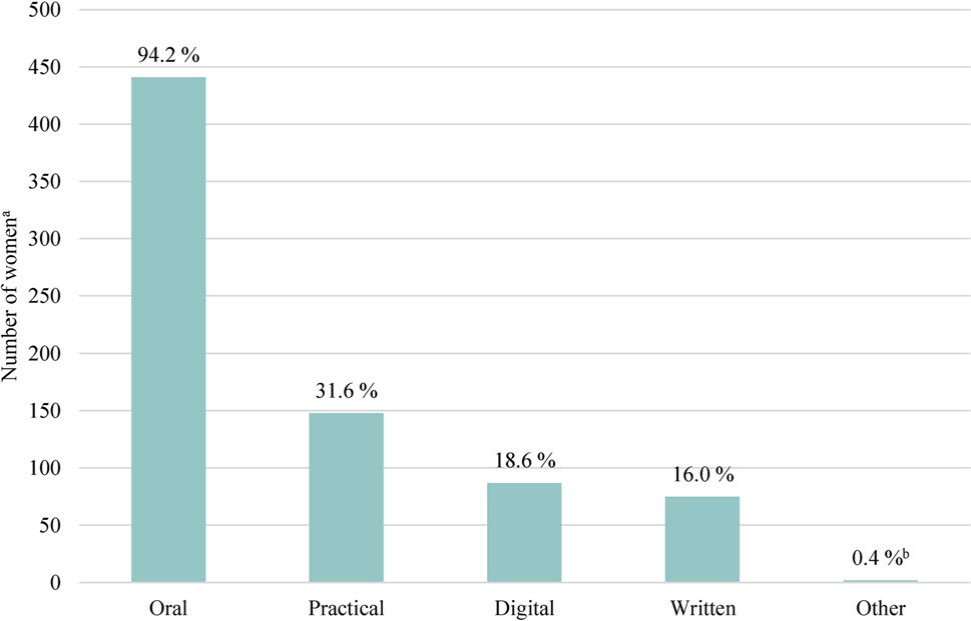
Form in which information about antenatal colostrum expression was received from healthcare personnel. ^a^ Only participantswho responded “Yes” to the question “Did you receive information about antenatal colostrum expression from healthcare personnel?” received this question (*n* = 468). ^b^ Respondents could tick multiple answers. Total percentage therefore exceeds 100%

Figure 4 illustrates the form in which information about ACE was received from healthcare personnel. Nearly all women reported receiving oral information, either as the only form or in combination with other forms. Approximately one-third received practical information, for example via demonstration with a model. Fewer reported receiving digital information, such as from social media or hospital websites, or written information.

**Fig. 5:**
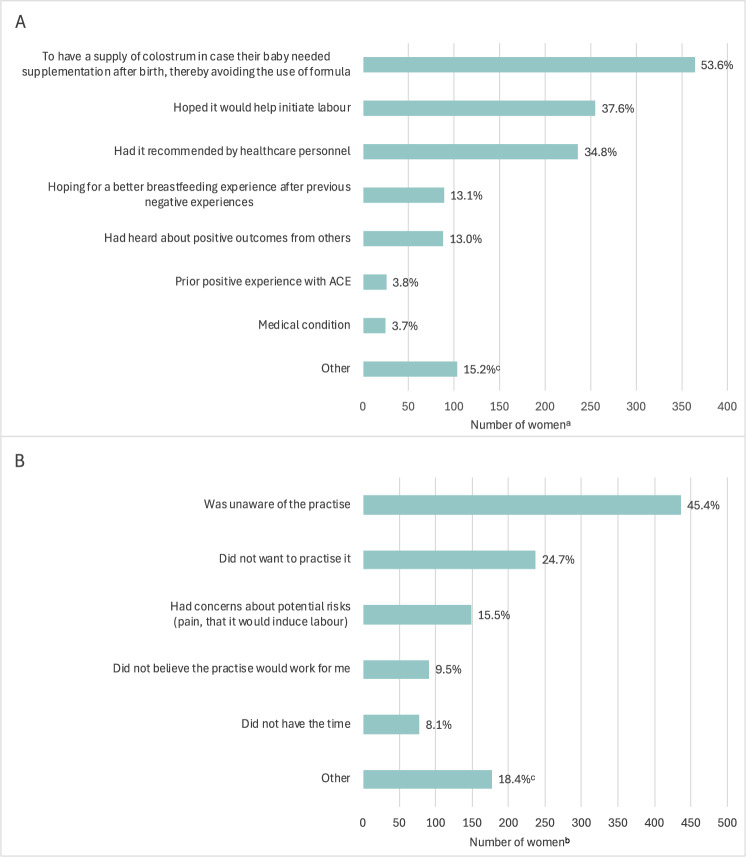
Reasons women reported (**A**) practising antenatal colostrum expression or (**B**) not practising antenatal colostrum expression. a Only participants who responded “Yes” to the question “Did you practise antenatal colostrum expression?” received this question (*n* = 679). b Only participants who responded “No” to the question “Did you practise antenatal colostrum expression?” received this question (*n* = 961). c Respondents could tick multiple answers. Total percentage therefore exceeds 100%

Figure 5 (A) visualizes reasons women reported for practising ACE. The most frequently stated reason, cited by just over half of respondents, was to have a supply of colostrum in case their baby needed supplementation after birth, thereby avoiding the use of formula. Over one-third of women stated they had practised ACE because it had been recommended by healthcare personnel or because they hoped it would help initiate labour. Other reasons included hearing positive outcomes from others or hoping for a better breastfeeding experience after previous negative experiences. Fewer women cited a medical condition or prior positive experience with ACE.

 Figure 5 (B) shows reasons why women did not practise ACE. Nearly half reported that they were unaware of the practice. One-quarter said they did not want to practise it. A significant number did not practise ACE due to concerns about potential risks. Nearly one in ten either did not have the time or believed the practise would not work for them (Fig. 5).

## Discussion

The study investigated whether, and in which form, pregnant women received information about ACE, as well as the characteristics of those who practised it compared to those who did not. Results showed that two thirds of women received information about ACE during pregnancy. A quarter received information from healthcare personnel, while twice as many obtained it from other sources. Most information from healthcare personnel came from midwives and was delivered orally. Women who had practised the method were more likely to have known about it before pregnancy, to have received information from healthcare personnel or other sources, and/or to have had it recommended by healthcare providers. Practising the method was associated with being younger and a first-time mother. The most common reason for practising ACE was to avoid the use of infant formula; the most common reason for not practising it was lack of awareness. Findings are discussed in relation to self-efficacy theory [27].

Slightly more than one third of women were aware of ACE before pregnancy. Awareness and information from healthcare personnel were associated with practising the method. International studies have shown varying levels of awareness [17, 20, 23, 24]. Women reported that evidence-based information was a key factor in their decision making [24], and several studies show that education and guidance from healthcare personnel increase likelihood of practising ACE [12, 37]. Although two thirds of women in this study received information, roughly one quarter received it from healthcare personnel, most often by midwives. This is consistent with findings from other studies, where women who received information from healthcare personnel, primarily received it from midwives or lactation consultants [4, 11, 16]. Lack of national guidelines [32] and provider knowledge [10, 17, 38] may explain why many women were not informed by healthcare personnel. Still, one in five women were recommended to practise ACE by a provider, and over two in five had practised it. Previous studies confirm that professional recommendations increase the likelihood of practising ACE [12, 24], suggesting the importance of professional support in relation to strengthening breastfeeding self-efficacy [27].

Twice as many women received information from non-healthcare sources, particularly Ammehjelpen (the Norwegian breastfeeding mother-to-mother support group) and social media. Other studies also found that women used internet and social media as source of information about ACE [16, 17, 22]. These sources can broaden access but raise questions about accuracy and consistency. Women who practised ACE more often perceived the information they received as sufficient. In previous studies women reported uncertainty or lack of guidance [22, 23]. Supportive follow-up may build self-efficacy [39]. More research is needed to understand which approaches best prepare women to practise the method.

Most women received oral information from healthcare personnel, and around one third were offered practical demonstrations. Some women have reported preferring to practise in private due to feelings of embarrassment [19], while others described how practical demonstrations by healthcare personnel facilitated increased partner involvement [20]. These findings highlight that emotional safety and supportive communication are important for women to feel comfortable with practising ACE [23] and for building self-efficacy [27]. Few women in existing research reported receiving digital or written information, although digital tools have shown potential as effective learning resources [38]. Instructional videos and peer-based learning approaches have been positively evaluated in earlier studies [10, 19]. Although some hospitals in Norway have published information about ACE on their websites, e.g. [34], the available content is often limited.

Practising ACE was associated with being younger and a first-time mother, unlike findings in some Australian studies [12, 26]. This may reflect that first time mothers lack previous breastfeeding experience, which is strongly linked to breastfeeding self-efficacy [39]. Pregnancy represents a crucial period for preparing women for breastfeeding and for strengthening their breastfeeding self-efficacy [40]. ACE may help women prepare for breastfeeding [4, 17]. Many women would consider practising ACE to prepare for breastfeeding [24] and first time mothers may be especially motivated [20]. Although ACE is often highlighted for women with diabetes [10], few participants cited medical conditions. Most practised the method to prepare a colostrum supply and avoid formula use, consistent with findings from other countries [24, 26]. Breast milk is known to benefit newborns [1, 3], and reducing non medically indicated formula use is a public health goal [41, 42]. Although some evidence suggests ACE may reduce formula use among women with diabetes [12], findings are inconsistent [14, 17], and more research is needed.

A third of women who practised the method said it was recommended by healthcare personnel. Previous research has also found that women who practised ACE, had it recommended by healthcare personnel [24]. Negative past experiences also motivated some women, possibly to build self-efficacy for future breastfeeding [10, 39]. Other motivators included seeing others succeed and encouragement from family and friends [26, 27], which can be related to two of the four sources that influence self-efficacy, namely vicarious experiences and verbal persuasion [27]. Few women cited previous positive experience, likely due to the high proportion of first-time mothers, though this may increase as awareness grows [8, 9].

Some women hoped the method would induce labour, while others avoided it for the same reason. Although oxytocin is involved in both processes, studies show ACE does not cause labour when initiated from week 34–37 [8, 9, 11]. Misconceptions may stem from lack of knowledge among both women and healthcare personnel [10]. A few women felt the method would not work for them. Confidence may increase with support and success [9, 11, 15, 16, 26]. Others did not want to practise ACE or lacked the time. Previous studies have demonstrated that some women perceived ACE as time consuming [22, 23], while others found it worthwhile [15, 16, 21]. 

Nearly half of the women who did not practise ACE said they had never heard of it. The lack of national guidelines [32] may contribute to inconsistencies in who receives information. Without awareness, they had no opportunity to make an informed choice. According to the Norwegian Patients’ Rights Act [43], individuals have the right to information necessary to make decisions regarding their health. Women need evidence-based information from healthcare personnel to make informed choices [10, 44].

### Strengths and limitations

The study had some important strengths and limitations. The large sample size enhanced the reliability of the findings [45]. The use of a structured and piloted questionnaire ensured consistency in data collection [46]. However, the questionnaire was not validated, which may have affected the measurement accuracy, and the study's results may be affected by instrument bias. These methodological constraints should be considered when interpreting the results and their generalizability [47]. The study population is not representative of all pregnant women in Norway. Recruitment through self-selection and social media may have introduced selection bias [46], as women with particular interest in breastfeeding may have been more inclined to participate, and the Norwegian-only format likely excluded women with limited language proficiency, particularly immigrants [48]. Not collecting detailed demographic characteristics strengthened participant anonymity and reduced the risk of indirect identification. At the same time, this choice represents a limitation as it reduces the possibility of conducting subgroup analyses. A deliberate choice was made to apply descriptive rather than advanced statistical modelling, in line with the exploratory aim of the study; however, future research may benefit from more complex analyses to further investigate associations and predictors.

## Conclusion

Women who received information about ACE primarily obtained this information from other sources than healthcare personnel. Younger and first-time mothers were more likely to practise ACE, often with the aim of reducing formula use. Women who engaged in ACE were more likely to have received information from healthcare personnel, most commonly by midwives through oral communication. The most frequent reason for not practising ACE was lack of awareness. The findings indicate that improved access to information, particularly from healthcare providers, may encourage more women to consider ACE, and highlight the need for clear national guidelines to ensure consistent and evidence-based communication about ACE across maternity care settings. Further research is warranted to explore whether ACE can reduce formula use, increase exclusive breastfeeding rates and prolong breastfeeding duration.

## Supplementary Information

Additional File 1.

## Data Availability

The study was conducted in accordance with UiT The Arctic University of Norway’s guidelines for data management. In line with these guidelines, the data file will be deleted after the study is published.

## Abbreviation

ACE: Antenatal colostrum expression
