# Supplementary material for: Information and practices of antenatal colostrum expression: a cross-sectional study of pregnant women in Norway
Source: BMC Pregnancy Childbirth. 2026 May 21;26:820. doi: 10.1186/s12884-026-09035-y (PMC13412171; doi:10.1186/s12884-026-09035-y)
Supplement: Supplementary file 1 — Additional File 1. [file 12884_2026_9035_MOESM1_ESM.pdf]

Nettskjema

Antenatal colostrum expression

Part 1: Background characteristics

Are you over 18 years of age?

Yes
No

Did you give birth in Norway between 1 October 2023 - 30 September 2024?

Yes
No

Did you give birth after gestational week 37+0?

Please answer based on your birth between 1 Oct 2023 and 30 Sept 2024

Yes
No

How old are you?

18-24
25-29
30-34
35-39
40 or older

What is your highest completed level of education?

Primary/secondary/sixth form colleges
University, 1-4 years
University, more than 4 years

Which health region did you belong to?

Please answer based on your birth between 1 Oct 2023 and 30 Sept 2024

Northern Norway
Central Norway
Western Norway
South-East Norway

Were you a primipara or a multipara?

Please answer based on your birth between 1 Oct 2023 and 30 Sept 2024

Primipara
Multipara
Other

During which of these two periods did you give birth?

Please answer based on your birth between 1 Oct 2023 and 30 Sept 2024

01.10.23 - 31.03.24
01.04-24 - 30.09.24

Part 2: Antenatal colostrum expression

Did you practise antenatal colostrum expression?

Antenatal colostrum expression refers to expressing breast milk during pregnancy using a repetitive technique with your hand, beginning from gestational week 36+0 at the earliest. The use of an electrical pump is not considered antenatal colostrum expression.

- Yes
- No
- Other

Why did you practise antenatal colostrum expression?

Tick one or more alternatives

- Had it recommended by a healthcare personnel
- Medical condition
- Hoped it would initiate labour
- Had heard about positive outcomes from others
- Prior positive experience with ACE
- Hoping for a better breastfeeding experience after previous negative experiences
- To have a supply of colostrum in case my baby needed supplementation after birth, thereby avoiding the use of formula
- Other

Why did you not practise antenatal colostrum expression?

Tick one or more alternatives

- Was unaware of the practise
- Had concerns about potential risks (pain, that it would induce labour)
- Did not have the time
- Did not believe the practise would work for me
- Did not want to practise it
- Other

To what extent were you satisfied with antenatal colostrum expression?

- To a small extent
- To some extent
- To a large extent
- To a very large extent
- Other

Part 3: Information about antenatal colostrum expression

Were you aware of antenatal colostrum expression before becoming pregnant?

Please answer based on your birth between 1 Oct 2023 and 30 Sept 2024

- Yes
- No
- Other

Who did you mainly visit for prenatal consultations?

Please answer based on your birth between 1 Oct 2023 and 30 Sept 2024

- Midwife
- General practitioner (GP)
- A combination of services (midwife, GP, maternity wards or outpatient clinics)
- Did not attend prenatal consultations
- Other

Did you receive information about antenatal colostrum expression from healthcare personnel?

Please answer based on your birth between 1 Oct 2023 and 30 Sept 2024

- Yes
- No
- Other

Did you receive information about antenatal colostrum expression during prenatal consultations or courses from healthcare personnel in municipal services?

Please answer based on your birth between 1 Oct 2023 and 30 Sept 2024

- Yes
- No
- Did not attend prenatal consultations
- Other

Did you receive information about antenatal colostrum expression during prenatal consultations or courses from private healthcare providers?

Please answer based on your birth between 1 Oct 2023 and 30 Sept 2024

- Yes
- No
- Did not visit private healthcare providers
- Other

Did you receive information about antenatal colostrum expression at the maternity ward prior to the birth?

Please answer based on your birth between 1 Oct 2023 and 30 Sept 2024

- Yes, during an admission/ultrasound/outpatient appointment at the maternity ward/outpatient clinic prior to the admission related to the birth
- Yes, during the admission related to the birth
- No, I did not receive information about ACE during an admission/ultrasound /outpatient appointment at the maternity ward/outpatient clinic
- Was not admitted/at an ultrasound or outpatient appointment prior to the birth
- Other

Which type of healthcare personnel did you receive information about antenatal colostrum expression from?

Tick one or more alternatives

- Midwife
- General practitioner
- Gynecologist
- Nurse
- Other

In which form did you receive information about antenatal colostrum expression from healthcare personnel?

Tick one or more alternatives

- Oral
- Written
- Practical (for example via demonstration with a model)
- Digital (for example healthcare personnel on social media or hospital websites)
- Other

Were you recommended by healthcare personnel to practise antenatal colostrum expression?

Please answer based on your birth between 1 Oct 2023 and 30 Sept 2024

- Yes
- No
- Other

To what extent did you feel that the information you received from healthcare personnel was sufficient to be able to potentially practise antenatal colostrum expression?

Please answer based on your birth between 1 Oct 2023 and 30 Sept 2024

- To a small extent
- To some extent
- To a large extent
- To a very large extent
- Other

Did you receive information about antenatal colostrum expression from other sources than healthcare personnel?

Please answer based on your birth between 1 Oct 2023 and 30 Sept 2024

- Yes
- No
- Other

Which other sources than healthcare personnel did you receive information about antenatal colostrum expression from?

Tick one or more alternatives

- Social media
- Influencer
- Ammehjelpen (the Norwegian breastfeeding mother-to-mother support group)

- Family member
- Friend
- Book
- TV-show
- Other

Which social media platforms did you receive information about antenatal colostrum expression from?

Tick one or more alternatives

- Facebook
- Instagram
- Snapchat
- TikTok
- Other

To what extent did you feel that the information you received from other sources than healthcare personnel was sufficient to be able to potentially practise antenatal colostrum expression?

Please answer based on your birth between 1 Oct 2023 and 30 Sept 2024

- To a small extent
- To some extent
- To a large extent
- To a very large extent
- Other
